# The National Student Performance Examination and the quality of Brazilian higher education in health

**DOI:** 10.1590/1518-8345.5714.3534

**Published:** 2022-05-30

**Authors:** Bruno Luciano Carneiro Alves de Oliveira, Fabiana Alves Soares, Aida Patrícia da Fonseca Dias Silva, Carlos Leonardo Figueiredo Cunha, Jouhanna do Carmo Menegaz, Kênia Lara da Silva

**Affiliations:** 1 Universidade Federal do Maranhão, São Luís, MA, Brasil.; 2 Universidade Federal do Pará, Belém, PA, Brasil.; 3 Universidade do Estado de Santa Catarina, Chapecó, SC, Brasil.; 4 Universidade Federal de Minas Gerais, Belo Horizonte, MG, Brasil.

**Keywords:** Educational Measurement, Teaching Institutions, College Education, Health Education, Health Sciences, Privatization, Avaliação Educacional, Instituições de Ensino, Educação Superior, Educação em Saúde, Ciências da Saúde, Privatização, Evaluación Educacional, Instituciones de Enseñanza, Educación Universitaria, Educación en Salud, Ciencias de la Salud, Privatización

## Abstract

**Objective:**

to analyze higher education in the health area in Brazil according to the results of the students’ performance in the 2019 National Student Performance Examination.

**Method:**

a cross-sectional and retrospective study with a quantitative approach and online data referring to 192,715 students from 3,712 health courses, collected in 2020. The students’ performance was expressed in concepts, ordered on a scale of one to five. Box-plots were prepared, prevalence values of the concepts were estimated, and the differences were evaluated through statistical tests (α=5%) according to the administrative and management characteristics, as well as those of the cities where the courses were offered.

**Results:**

there was a satisfactory level of higher education in health (70.0% with a score ≥3). The Medicine courses were the most satisfactorily evaluated while Speech Therapy and Nursing obtained the worst scores. Public, face-to-face and university education were associated with better teaching quality. Lower levels were found in the North region states, in small towns, outside metropolitan areas and in those under private management.

**Conclusion:**

heterogeneity in the quality of higher education across the health courses was verified, indicating its association with the characteristics of the teaching institutions and with the infrastructure of the cities where the courses are offered, pointing out challenges to the quality of health education in Brazil.

## Resumo

**Objetivo:**

analisar o ensino superior da área da saúde no Brasil segundo os resultados do desempenho dos estudantes no Exame Nacional de Desempenho dos Estudantes de 2019.

**Método:**

estudo transversal, retrospectivo, de abordagem quantitativa com dados *on-line* referentes a 192.715 estudantes de 3.712 cursos da saúde, coletados em 2020. O desempenho dos estudantes foi expresso em conceitos, ordenados em escala de um a cinco. Foram elaborados *box-plot* e estimadas prevalências dos conceitos e avaliadas diferenças segundo as características administrativas, de gestão e das cidades sede dos cursos por meio de teste estatísticos (α=5%).

**Resultados:**

observou-se nível satisfatório do ensino superior de saúde (70,0% com conceito ≥3). Cursos de medicina foram os mais satisfatoriamente avaliados, fonoaudiologia e enfermagem os piores. O ensino público, presencial e em universidades estiveram associados à maior qualidade do ensino. Níveis mais baixos foram verificados nos estados da região Norte, nas cidades pequenas e fora de áreas metropolitanas e naqueles sob gestão privada.

**Conclusão:**

verificou-se heterogeneidade na qualidade do ensino superior entre os cursos de saúde, indicando sua associação com características das instituições de ensino e à infraestrutura das cidades sedes dos cursos, apontando desafios para a qualidade da formação em saúde no Brasil.

## Resumen

**Objetivo:**

analizar la educación superior en el área de la salud en Brasil en función de los resultados del desempeño de los estudiantes en el Examen Nacional de Rendimiento de los Estudiantes de 2019.

**Método:**

estudio transversal, retrospectivo con enfoque cuantitativo con datos *online* referentes a 192.715 estudiantes de 3.712 carreras de salud, recolectados en 2020. El desempeño de los estudiantes se expresó en conceptos, ordenados en una escala de uno a cinco. Se elaboraron *box-plots* y se estimaron y las prevalencias de los conceptos y se evaluaron las diferencias según las características administrativas, de gestión y de las ciudades sede de las carreras a través de pruebas estadísticas (α=5%).

**Resultados:**

se observó un nivel satisfactorio de educación superior en salud (70,0% con concepto ≥3). Las carreas de Medicina obtuvieron las mejores calificaciones, las de Fonoaudiología y Enfermería las peores. Se asoció la educación pública, presencial y universitaria con una educación de mayor calidad. Los niveles más bajos se encontraron en los estados de la región Norte, en los pueblos pequeños y fuera de las áreas metropolitanas, y en las carreras de gestión privada.

**Conclusión:**

se verificó que había heterogeneidad en la calidad de la educación superior en las carreras de salud, lo que indica que hay una asociación entre las características de las instituciones educativas y la infraestructura de las ciudades donde se dictan las carreras, y señala que la calidad de la educación en salud en Brasil enfrenta desafíos.

**Highlights**

(1) The results indicate satisfactory levels in higher education students.

(2) Heterogeneity in the quality of higher education among the health courses.

(3) Association of the ENADE score with characteristics of the institutions and host cities.

(4) Heterogeneity in teaching quality both between and within regions of the country.

(5) Need to order regulatory measures to correct disparities.

## Introduction

The evaluation of higher education represents an important stage in the analysis of the quality standards of the education offered by national education systems, individual institutions and undergraduate and graduate programs in the several knowledge areas in different countries^1^.

The literature reveals that the first proposals for the implementation of higher education evaluation policies were elaborated in the United States of America (USA) and England, and later replicated by other countries. However, appropriation and adherence to these policies and guidelines did not occur in a linear and homogeneous way across the countries, with different evaluation dynamics occurring^2^.

In the USA, the evaluation and accreditation system for Higher Education Institutions (HEIs) is decentralized, specialized by knowledge area and under the responsibility of accreditation agencies (non-profit or non-governmental private companies). In the United Kingdom, the process is state-owned, but respects the autonomy of the HEIs. In Spain, the evaluation focus is on the course and not on the HEI, and the character is mixed in Mexico: mandatory evaluation for public HEIs and voluntary for private HEIs^3^ ^-^ ^6^.

In Brazil, the first initiative to evaluate higher education dates back from 1995, with the implementation of the National Examination of Courses, also known as *Provão*. Subsequently, the Higher Education Center and the Evaluation of the Teaching Conditions were created^7^ ^-^ ^8^. Currently, the country has a comprehensive system to evaluate the quality of higher education, based on different methods and instituted by Law No. 10,861/2004: the National System for the Evaluation of Higher Education (*Sistema Nacional de Avaliação do Ensino Superior*, SINAES). This is linked to the Anísio Teixeira National Institute of Educational Research Studies (*Instituto Nacional de Estudos e Pesquisas Educacionais*, INEP) belonging to the Brazilian Ministry of Education, and aims at ensuring a national process to evaluate higher education in the country^7^ ^-^ ^8^.

SINAES has three components: evaluation of the HEIs, of the undergraduate courses and of the students’ academic performance. The first two seek to attest to the quality of the teaching offered based on a matrix of indicators about the faculty, the didactic-pedagogical organization and the course infrastructure; these evaluations are carried out *in locus* by teams of professors external to the HEIs. The third component is the assessment test of the undergraduate students’ learning outcomes and occurs through the National Student Performance Examination (*Exame Nacional de Desempenho do Estudante*, ENADE)^7^.

The three components of SINAES are expressed through concepts, ordered on a scale with five levels^7^, and allow knowing the mode of operation and the quality of the courses and HEIs throughout Brazil.

Specifically, ENADE seeks to assess the students’ performance against the programmatic contents provided for in the general training and in the curricular guidelines of their respective undergraduate areas. Thus, it allows verifying the students’ skills, an approximation to the evolution of knowledge, the competences and concatenation to understand topics beyond those specific to their profession, articulating them to other knowledge areas in the face of the Brazilian and world reality^9^. 

ENADE is applied annually since 2004. During this period, it has already undergone several modifications that improved this evaluation method and guaranteed the ability to reveal the quality stage of higher education from the students’ performance^1^ ^-^ ^2^ ^,^ ^7^. The Examination is applied periodically to students from different knowledge areas^9^; these areas take turns each year to do the test^9^. In 2019, in addition to the courses in the Engineering, Agronomy, Architecture and Urbanism, and Zootechnics areas, as well as some technological courses, ten courses in the health area were evaluated, namely: Biomedicine, Physical Education, Nursing, Pharmacy, Physiotherapy, Speech Therapy, Medicine, Veterinary Medicine, Nutrition and Dentistry. They comprised 49.5% of the students and 44.8% of the courses evaluated^10^.

Despite being a well-known evaluation and used to measure the quality of higher education, in general, the ENADE data are more used for comparisons between courses of the same undergraduate degree, generating more specific and isolated debates about the teaching quality in each course. This condition contributes little to a broader discussion about the general situation of higher education in Brazil and about the influences of the courses’ structural, geographical, pedagogical and organizational characteristics. In this sense, it is necessary to produce diverse evidence on the quality of higher education considering the Brazilian socioeconomic and geographical differences. 

Thus, similarly to other countries, in Brazil there is an expansion of higher education in health with characteristics of privatization, inland-reach and intense use of Education at a Distance technologies^1^ ^,^ ^11^. This movement responds to a series of policies that contributed to the growth of public and private education in all regions and in cities of different population sizes, making it a challenge to understand the impact of these actions on the current quality of higher education in Brazil^12^. 

In the case of the health sector, the ENADE results can be a useful indicator of the quality of the training of the professionals who will work in the Brazilian health system, and the quality of this training can be partially reflected in the functioning and resoluteness of the health services. Therefore, the analysis of ENADE data allows discussing the general social benefits involved in expanding access to higher education and its consequences for the health system^11^. In addition, due to its regulatory character, ENADE influences the quality, reputation and survival of the HEIs in the offer of different courses^8^. 

Thus, this study sought to analyze higher education in the health area in Brazil according to the results of the students’ performance in the 2019 National Student Performance Examination. 

## Method

### Type of study, period and locus

This is a cross-sectional and retrospective study with a quantitative approach, based on secondary data from the 2019 National Examination of Student Performance (ENADE), available online in INEP’s electronic system, belonging to the Brazilian Ministry of Education, and collected in November 2020. Communication of the results followed the STROBE tool guidelines.

### Population and selection criteria 

The students attending the Bachelor’s Degree Courses who expected to complete the course by July 2020 or with 80% or more of the minimum course load completed by the end of the Examination enrollment period in that year participated in ENADE 2019^9^. For this research, only data from the undergraduate courses in the health area were included, namely: Biomedicine, Physical Education, Nursing, Pharmacy, Physiotherapy, Speech Therapy, Medicine, Veterinary Medicine, Nutrition and Dentistry; excluding the other courses not belonging to this area.

In ENADE 2019, 192,779 students (91.4% of those enrolled) participated in 3,748 undergraduate health courses in the country. For this study, only the courses that obtained some course concept were analyzed: 3,712 courses and their 192,715 students. The reason why some courses did not obtain a concept is that those with less than two participants were considered as “No Concept (NC)” courses since, according to §9 of article 5 of Law No. 10,861, dated 04/14/2004, this condition is necessary to preserve the student’s identity^7^ in addition to the courses with a mean performance equal to zero (also NC, as they are not considered in the calculation)^9^.

### Study variables

The performance of the students attending each of the participating courses is expressed through concepts, ordered on a scale with 5 (five) levels^7^. The official ENADE database according to undergraduate courses and administrative, management and contextual characteristics of the HEIs can be found in the INEP website. For this research, the following variables were described: undergraduate course, teaching modality (face-to-face or at a distance - EaD), number of participating students, concept in ENADE, type of academic organization [University, University Center, College and Federal Education, Science and Technology Institute (*Instituto Federal de Educação, Ciência e Tecnologia*, IFECT)], administration (public or private), type of public management (federal, state and municipal), Federation Unit (FU), macro-region of the country (Northeast, North, Midwest, Southeast and South), population size of the city where the course is offered (small, medium and large) and location in the Metropolitan Region (MR)^13^.

### Data treatment, analysis and statistics

The data were organized in Microsoft Excel^®^ and then transferred to the Stata^®^ software, version 14.0. The absolute and relative frequencies of the courses evaluated and the prevalence of the course concepts in ENADE were verified. Box-plots were prepared with the concepts of the undergraduate courses according to the type of administration (public or private) and public management (federal, state and municipal). For the FU and region of the country variables, the prevalence values of unsatisfactory concept (sum of grades 1 and 2) of the undergraduate courses was estimated. Finally, according to the region of the country, the prevalence values of the course concepts were verified according to the administrative, management and contextual characteristics of the cities where courses evaluated are offered. Pearson’s Chi-square test was used to verify statistically significant differences in this last evaluation stage. The significance level adopted was 5% (*α=0.05*).

### Ethical aspects

In line with the rules in force in Brazil on ethics in research with human beings, research studies carried out with aggregates of secondary data available online and with open and public access, which preserve anonymity of those investigated, do not need to be submitted for consideration by any Research Ethics Committee (Resolution No. 510/16 of the National Health Council)^14^. Therefore, this research was not submitted to the appreciation of any Research Ethics Committee.

## Results

In this study, the ENADE 2019 results were described for 192,715 students and 3,712 undergraduate health courses. The Nursing undergraduate course was the one that included the largest number of participants (38,270; 19.9%) and courses evaluated (793; 21.4%); Speech Therapy had the lowest representation (2,426 students; 2.0% of the courses).

Table 1 shows that most of the courses evaluated were organized by universities (43.2%) and offered by private HEIs (81.7%). Among the public HEIs, most were under federal management (63.6%). There was predominance of courses in the Southeast region (43.5%) and in large cities (79.1%).

In the health area, courses with concept 3 (40.1%) prevailed and only 6.4% of the courses obtained the highest grade possible The proportions of concept 3 varied from 13.7% in Speech Therapy to 46.5% for Physical Education. The Medicine course obtained a higher proportion of concept 4 (38.8%) and Speech Therapy presented a higher proportion of concept 2 (30.1%). The Biomedicine (33.2%) and Nursing (33.9%) courses presented the highest proportions of concept 2. Speech Therapy had the highest proportions of courses with concepts 1 (17.8%) and 5 (13.7%) (Table 1).

In face-to-face education, there was predominance of courses with concept 3 (40.1%), and courses with concept 2 (55.6%) prevailed in EaD. Whereas the courses offered by university centers (46.8%) and universities (36.2%) mostly presented concept 3, the college courses obtained concept 2 in 41.3%, and those offered by IFECT obtained concept 4 in 46.7% of the courses. The universities also presented a relevant proportion of courses with concept 4 (34.6%). In relation to the type of administration, in the public sector, courses with concepts 4 (48.1%) and 5 (27.2%) were more frequent, while concepts 3 (45.3%) and 2 (30.8%) were more frequent in institutions under private administration. In public education, federal courses predominated with concept 5 (33.7%), state courses with concept 4 (57.3%) and municipal courses with concept 2 (38.1%) (Table 1). 

Table 1 also shows that, among the regions of the country, courses with concept 2 were the majority in the North region (43.6%) and those with concept 3 in the other regions, with emphasis on the South (46.1%) and Southeast (42.9%). The North region also presented the highest proportion of concept 1 (13.1%) and the Northeast region, concept 5 (8.3%). Regarding the population size of the cities where the courses are offered, those with concept 3 predominated in large (40.2%) and medium-sized (40.7%) cities. Small cities presented higher proportions of courses with concepts 1 (12.6%) and 2 (32.4%). Concepts 4 (23.9%) and 5 (6.9%) were more recorded in large cities. The courses implemented outside the MR presented higher proportions of concepts 2 (27.3%) and 3 (40.5%).

**Table 1:** General characteristics and concepts of the undergraduate courses in the health area (n=3,712) in Brazil, according to the performance of their students (n=192,715) in ENADE 2019

Characteristics	N	%	ENADE concepts (%)				
			1	2	3	4	5
Undergraduate courses	3,712	100.0	4.7	26.2	40.1	22.6	6.4
Biomedicine	301	8.1	4.7	33.2	39.5	19.9	2.7
Physical Education	501	13.5	3.2	22.4	46.5	22.9	5.0
Nursing	793	21.4	6.8	33.9	39.1	16.5	3.7
Pharmacy	429	11.6	2.6	21.7	41.5	27.7	6.5
Physiotherapy	517	13.9	3.7	26.9	44.3	17.8	7.3
Speech Therapy	73	2.0	17.8	30.1	13.7	24.7	13.7
Medicine	232	6.2	5.6	7.8	35.8	38.8	12.0
Veterinary Medicine	215	5.8	9.8	27.4	35.8	20.5	6.5
Nutrition	414	11.1	1.9	25.4	37.7	25.1	9.9
Dentistry	237	6.4	2.1	24.1	39.2	27.9	6.7
**Teaching modality**							
Face-to-face	3,694	99.5	4.7	26.1	40.1	22.7	6.4
Education at a Distance	18	0.5	11.1	55.6	27.8	0.0	5.5
**Academic organization**							
University Center	1,021	27.5	4.0	30.5	46.8	17.4	1.3
College	1,074	28.9	8.5	41.3	39.7	9.2	1.3
IFECT^*^	15	0.4	0.0	13.3	26.7	46.7	13.3
University	1,602	43.2	2.6	13.6	36.2	34.6	13.0
**Administration**							
Public	681	18.3	2.1	6.0	16.6	48.1	27.2
Private	3,031	81.7	5.3	30.8	45.3	16.9	1.7
**Public management**							
Federal	433	63.6	0.2	2.1	13.9	50.1	33.7
State	185	27.2	0.5	4.3	16.8	57.3	21.1
Municipal	63	9.2	19.1	38.1	34.9	7.9	0.0
**Macro-region of the country**							
North	259	7.0	13.1	43.6	28.6	13.1	1.6
Northeast	842	22.7	4.4	33.0	35.2	19.1	8.3
Midwest	351	9.4	7.4	28.5	36.5	23.4	4.2
Southeast	1,614	43.5	3.9	23.8	42.9	22.8	6.6
South	646	17.4	2.2	15.3	46.1	30.0	6.4
**Population size of the city^†^ where the course is offered**							
Small size	182	4.9	12.6	32.4	36.3	14.8	3.9
Medium size	594	16.0	5.9	30.5	40.7	18.5	4.4
Large size	2,936	79.1	4.0	25.0	40.2	23.9	6.9
**Metropolitan Region (MR)**							
Yes	1,921	51.8	4.1	25.3	39.7	23.3	7.6
No	1,791	48.2	5.3	27.3	40.5	21.9	5.0

Source: INEP, 2020. ^*^ *Instituto Federal de Educação, Ciência e Tecnologia*; ^†^Small size: <50,000 inhabitants and Demographic Density (DD) <80 inhabitants/km^2^; Medium size: From 50,000 to 100,000 inhabitants or DD ≥80 inhabitants/km^2^; and Large size: >100,000 inhabitants

**Figure 1:**
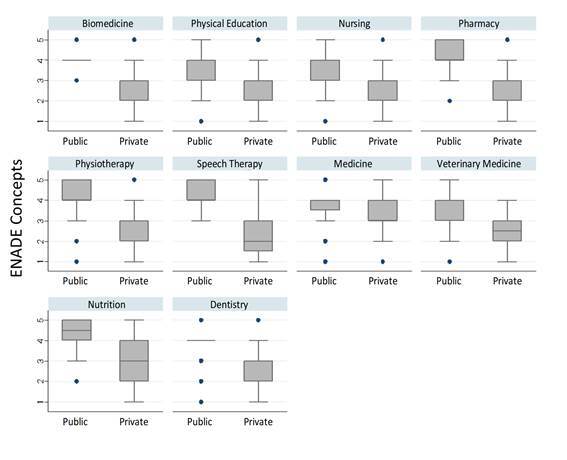
Box-plots with the concepts of the undergraduate courses in the health area in Brazil (n=3,712) by type of public or private administration, according to the performance of their students (n=192,715) in ENADE 2019

Source: INEP, 2020

The median of the course concept aggregating all the undergraduate courses was 3 (2-4). The highest median was in Medicine: grade 4 (3-4). All other courses presented a median of 3, with the lowest interquartile range for Biomedicine and Nursing (2-3) and the highest for Pharmacy (3-4). Systematically, the medians of the concepts of all the courses were higher for those under public management than for the private ones, with greater disparity for the Speech Therapy, Pharmacy, Physiotherapy, Nutrition and Dentistry courses, and lower for Medicine and Veterinary Medicine (Figure 1). 

**Figure 2:**
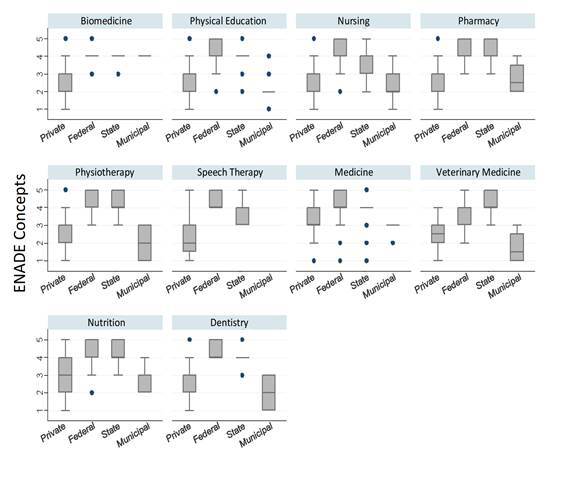
Box-plots with the concepts of the undergraduate courses in the health area in Brazil (n=3,712) by type of federal, state, municipal or private management, according to the performance of their students (n= 192,715) in ENADE 2019

Source: INEP, 2020

It was verified that courses under federal and state public management obtained better concepts, predominantly for Physical Education, Nursing, Speech Therapy, Medicine and Dentistry. The courses under state management with the best concepts were those of Veterinary Medicine. The courses under municipal management presented the worst concepts among those of public administration and, even when compared to those of private management, the Physical Education, Physiotherapy, Veterinary Medicine and Dentistry courses under municipal management were even worse evaluated (Figure 2).

The prevalence of unsatisfactory concept was 30.9% (n=1,148), being higher for Speech Therapy (48.0%), Nursing (40.7%), Biomedicine (37.9%) and Veterinary Medicine (37.2%) and lower for Medicine (13.4%) and Pharmacy (24.2%). Important differences were identified in the prevalence of unsatisfactory concept according to the FUs and regions of the country, with the worst situation observed in the North (56.8%) and the best in the South (17.5%). In general, this distribution pattern of unsatisfactory concept was repeated for most of the courses (Biomedicine, Physical Education, Nursing, Pharmacy, Physiotherapy, Nutrition and Dentistry). For the Speech Therapy and Medicine courses, the Midwest region presented worse estimates than the Northeast. The Medicine courses in the Northeast and South regions presented the lowest prevalence of unsatisfactory concept (Table 2).

**Table 2:** Prevalence of unsatisfactory concept (sum of grades 1 and 2; n=1,148) of the undergraduate courses in the health area in Brazil (n=3,712) by Federation Unit (FU), according to the performance of their students (n=192,715) in ENADE 2019

FU^*^	Undergraduate Courses in the Health Area										
	BM^†^	PE^‡^	NUR^§^	PHA^||^	PHY^¶^	ST^**^	MED^††^	VM^‡‡^	NUT^§§^	DENT^||||^	Total
**North**	63.0	27.3	65.6	58.8	40.0	100.0	47.1	52.9	61.9	63.6	56.8
ACRE	18.2	9.1	18.2	9.1	9.1	9.1	9.1	0.0	9.1	9.1	61.1
AMAZONAS	13.2	2.6	18.4	10.5	7.9	7.9	5.3	7.9	15.8	10.5	64.4
AMAPÁ	8.3	0.0	33.3	8.3	16.7	0.0	0.0	0.0	16.7	16.7	66.7
PARÁ	17.7	8.8	32.3	11.8	2.9	5.9	0.0	5.9	11.8	2.9	51.5
RONDÔNIA	7.7	0.0	34.6	23.1	3.9	7.7	7.7	7.7	0.0	7.7	47.3
RORAIMA	0.0	0.0	20.0	20.0	20.0	0.0	20.0	0.0	0.0	20.0	62.5
TOCANTINS	4.8	4.8	28.6	14.3	14.3	0.0	9.5	9.5	0.0	14.3	60.0
**Northeast**	55.2	32.1	46.9	31.3	40.0	47.4	5.1	22.6	42.0	21.8	37.4
ALAGOAS	5.0	15.0	35.0	5.0	15.0	0.0	0.0	5.0	10.0	10.0	41.7
BAHIA	16.2	7.1	28.3	8.1	22.2	2.0	0.0	0.0	12.1	4.0	41.8
CEARÁ	13.6	9.1	36.4	4.6	9.1	4.6	4.6	4.6	13.6	0.0	20.6
MARANHÃO	3.1	6.3	34.4	15.6	12.5	3.1	3.1	0.0	18.8	3.1	47.1
PARAÍBA	12.5	5.0	25.0	12.5	17.5	2.5	0.0	2.5	15.0	7.5	44.9
PERNAMBUCO	11.6	11.6	27.9	7.0	11.6	2.3	0.0	4.7	18.6	4.7	34.1
PIAUÍ	6.7	6.7	36.7	6.7	13.3	6.7	0.0	3.3	20.0	0.0	41.7
RIO GRANDE DO NORTE	17.7	0.0	41.2	5.9	5.9	5.9	5.9	0.0	17.7	0.0	30.9
SERGIPE	8.3	33.3	25.0	0.0	16.7	0.0	0,	8.3	8.3	0.0	30.0
**Midwest**	36.0	34.1	47.1	35.2	36.4	50.0	9.5	47.8	22.6	25.0	35.9
DISTRITO FEDERAL	4.8	4.8	38.1	14.3	14.3	4.8	4.8	9.5	4.8	0.0	24.1
GOIÁS	1.9	17.3	26.9	21.2	13.5	1.9	0.0	9.6	3.9	3.9	41.9
MATO GROSSO DO SUL	8.7	13.1	39.1	4.4	13.0	0.0	0.0	13.0	4.4	4.4	37.1
MATO GROSSO	16.7	6.7	30.0	13.3	10.0	0.0	3.3	3.3	10.0	6.8	38.5
**Southeast**	36.0	26.6	37.5	15.6	24.7	40.0	16.5	38.7	22.4	23.9	27.7
ESPIRITO SANTO	11.1	11.1	33.3	0.0	11.1	0.0	22.2	11.1	0.0	0.0	13.0
MINAS GERAIS	10.4	13.2	30.2	7.6	9.4	2.8	3.8	9.4	9.4	3.8	23.4
RIO DE JANEIRO	8.3	11.0	28.4	9.2	14.7	2.8	6.4	4.6	10.1	4.6	43.3
SÃO PAULO	12.6	17.5	24.7	4.9	13.5	1.8	1.4	1.4	9.0	5.8	26.6
**South**	4.4	13.6	21.4	13.9	26.4	35.3	5.3	33.3	7.5	18.8	17.5
PARANÁ	3.7	9.3	27.8	11.1	18.5	5.6	1.9	13.0	1.9	7.4	20.9
RIO GRANDE DO SUL	0.0	10.0	20.0	0.0	26.7	6.7	3.3	20.0	10.0	3.3	12.7
SANTA CATARINA	0.0	20.7	13.8	13.8	17.2	3.5	0.0	13.8	3.5	13.8	19.8
Total	37.9	25.6	40.7	24.2	30.6	48.0	13.4	37.2	27.3	26.2	30.9

Source: INEP, 2020. ^*^FU = Federation Unit; ^†^BM = Biomedicine; ^‡^PE = Physical Education; ^§^NUR = Nursing; ^||^PHA = Pharmacy; ^¶^PHY = Physiotherapy; ^**^ST = Speech Therapy; ^††^MED = Medicine; ^‡‡^VM = Veterinary Medicine; ^§§^NUT = Nutrition; ^||||^DENT = Dentistry

Among all the courses, variations were identified both within and between the regions. In the North region, the worst situation was for Speech Therapy, with 100% of the courses obtaining unsatisfactory concepts. In this region, the Biomedicine, Nursing, Nutrition and Dentistry courses had more than 60% of the courses with unsatisfactory concepts (Table 2).

Table 2 shows that, in the Northeast region, the Biomedicine course presented the highest proportion of unsatisfactory concept (55.2%), followed by Speech Therapy (45%) and Nursing (45%). In the Midwest region, the worst performance was observed for Speech Therapy, Nursing and Veterinary Medicine, with approximately 50% of the courses obtaining unsatisfactory concepts. In the Southeast region, nearly 40.0% of the Nursing, Veterinary Medicine, Biomedicine and Speech Therapy courses presented unsatisfactory concepts. In the South region, Speech Therapy and Veterinary Medicine are the courses with the highest prevalence of unsatisfactory concepts (≥33.3%).

The states with the worst evaluation of the students’ performance were Amapá (66.7%) and Amazonas (64.4%). The states of Espírito Santo (13.0%) and Paraná (12.7%) presented the lowest percentages of unsatisfactory concepts. Across the states, the Nursing courses generally had the highest estimates of unsatisfactory concept (Table 2).

Table 3 identifies the relationship between unsatisfactory concept and the structural and organizational characteristics of the HEIs and geographies where they are located in the country. Statistically significant differences (*p-value ≤ 0.02*) were verified between all the variables studied. Among the academic organization modalities, colleges presented the highest estimates, while universities and IFECT presented the lowest. Courses under private management also presented higher proportions of courses unsatisfactorily evaluated than the public ones in all regions of the country. In the public HEIs, the unsatisfactory concept varied from 1.5% in the South to 20.5% in the North and Midwest. Among the private HEIs, these rates were 66.3% in the North, 48.7% in the Northeast, 40.4% in the Midwest and 21.5% in the South. In the public administration HEIs, the courses under federal management presented the lowest proportions of unsatisfactory concept in all regions, and the municipal had the worst proportions, especially in the North (100.0%), Midwest (77.0%) and Southeast (56.3%) regions. State management presented the highest proportions for the Midwest region (25.0%).

**Table 3:** Prevalence of the unsatisfactory concept (sum of grades 1 and 2; n=1,148) of the undergraduate courses in the health area by Brazilian regions (n=3,712), according to the performance of their students (n=192,715) in ENADE 2019

Characteristics	Regions of the country					
	North (%)	Northeast (%)	Midwest (%)	Southeast (%)	South (%)	*p-value* ^*^
**Academic organization**						
University Center	47.8	42.7	38.4	31.8	20.7	*0.001*
College	75.2	56.6	55.8	39.4	37.3	
IFECT^†^	0.0	33.3	0.0	16.7	0.0	
University	38.4	10.7	15.5	18.8	10.6	
**Administration**						
Public	20.4	2.4	20.2	9.8	1.5	*0.001*
Private	66.3	48.7	40.4	30.4	21.5	
**Public management**						
Federal	9.5	1.5	5.6	0.7	0.0	*0.02*
State	16.7	4.1	25.0	4.2	0.0	
Municipal	100.0	0.0	77.0	56.3	16.8	
**Population size of the city^‡^ where the course is offered**						
Small size	90.9	30.8	72.4	42.3	28.8	*0.001*
Medium size	61.9	29.5	52.3	39.1	23.7	
Large Size	53.9	38.9	29.5	24.7	14.6	
**Metropolitan Region (MR)**						
Yes	58.3	38.2	26.3	28.3	11.3	*0.001*
No	55.4	36.1	45.3	27.3	26.4	

Source: INEP, 2020. ^*^Pearson’s Chi-square test; ^†^ *Instituto Federal de Educação, Ciência e Tecnologia*; ^‡^Small size: <50,000 inhabitants and Demographic Density (DD) <80 inhabitants^2^, Medium size: From 50,000 to 100,000 inhabitants or DD ≥80 inhabitants/km^2^, and Large size: >100,000 inhabitants

Table 3 also highlights the prevalence of unsatisfactory concept according to the size of the city where the course is offered. Among the courses offered in small municipalities in the North region, 90.9% were evaluated with unsatisfactory concept, as well as 61.9% in medium-sized municipalities and 53.9% in large cities. In the Midwest region, 72.4% of the courses whose headquarters are located in small municipalities also had unsatisfactory concept. In the South region, this rate was 28.8%. In relation to the headquarters being located in an MR, there were few differences in the same region, with the exception of the Midwest and South regions, where courses with unsatisfactory concepts outside the MR prevailed. However, the differences between the regions were significant: higher prevalence of unsatisfactory courses outside the MR in the North (55.4%) and Midwest (45.3%) regions.

## Discussion

This analysis pointed out important differences in the performance obtained in ENADE between the courses evaluated and their structural, organizational and geographical characteristics. In general, it was verified that most of the health undergraduate courses in Brazil presented a satisfactory performance level (70.0% with a score ≥3). However, only 6.4% of them reached the highest grade possible, also representing the significant distance from good quality education in the health sector in Brazil to the one offered in other middle- and high-income countries^15^.

In relation to ENADE 2016 (until then the last examination applied for undergraduate courses in the health area), the 2019 data show a reduction in the percentage of courses with satisfactory performance (73.0% with a concept ≥3 in 2016). From 2016 to 2019, only three undergraduate courses presented an improvement in the concepts, namely: Pharmacy, Medicine and Dentistry, especially Medicine, which achieved the greatest advance. On the other hand, Physiotherapy and Veterinary Medicine presented the greatest reductions^16^. Thus, mismatches are pointed out in the quality of the undergraduate courses in health over the last two ENADE examinations.

The students’ academic performance is a complex and multifactorial phenomenon, and can be influenced by four main groups of factors: student’s profile, faculty, management or teaching institutions, and external factors^17^ ^-^ ^19^. An international study found that the admission criteria, institutional factors and teaching and learning resources also have a significant relationship with the student’s performance and evaluation^20^.

In recent decades, the debate on these determinants of the performance of higher education students from different and specific knowledge areas has grown in Brazil. In 2019, despite the concentration of courses with good performance, it is pertinent to explore the disparities identified between them. The Medicine courses were the most satisfactorily evaluated and Speech Therapy and Nursing had the worst performance. 

Some explanations for these differences can be attributed to the characteristics of the students who present particular profiles depending on the course and to the knowledge area evaluated. In general, students attending Nursing and Speech Therapy courses need to work throughout their undergraduate studies and are subjected to physical and mental wear out resulting from the workday combined with the studies, as well as they tend to work in jobs little articulated with their study area, reducing the possibility of applying the knowledge arising from the undergraduate course^17^ ^-^ ^19^.

Socioeconomic inequalities also affect the higher education system by inducing social stratification, concentration of ethnic minorities and students with worse socioeconomic conditions in less prestigious courses and with lower wage returns^21^. These aspects have also influenced the ENADE results, especially in courses with higher female participation and low incomes among the students. In the case of Nursing, a previous study identified that more than half of the students associate work and study and, due to this double shift, they did not develop any activity beyond the mandatory ones foreseen in the curricular dynamics^22^. This characteristic negatively affects the student’s performance and the ENADE results. 

Another reason that explains the differences between courses is the high competition and selectivity levels in the admission processes for courses of greater social appreciation and prestige, such as Medicine. Competition to enter these courses can select a profile of students that tend to demand or pay for higher organizational levels in the HEIs consistent with the prospects of training and market they seek and aim for^19^ ^,^ ^23^. These processes can select students with greater chances of following the course and obtaining better grades in ENADE.

In the analysis with ENADE data from previous years, better performance was also observed in university graduates, who did not need to work during their undergraduate studies, attending public HEIs, especially federal, and students with better socioeconomic status^17^ ^,^ ^19^ ^,^ ^24^ ^-^ ^26^.

Despite these findings, the debate on the performance in ENADE should not be restricted to the students’ individual-personal characteristics, as structural, pedagogical and organizational factors of the courses exert an influence on the students’ performance. Thus, it is necessary to analyze the role of the institution, the teaching infrastructure and the cities where the undergraduate courses are offered, in order to understand their relationships with the general academic performance in the Exam.

In line with the findings of other studies, this research revealed that the ENADE result differs according to the structural, organizational and contextual factors of the courses evaluated^19^ ^,^ ^24^. The modalities of public education (especially federal), in-person and in universities or university centers were associated with higher education quality in the country. Lower performance levels were observed in EaD courses, for states in the North region, small cities and outside the MR.

More unsatisfactory performance was observed in courses under private management and in towns with smaller population sizes, which, in general, also corresponds to municipalities with worse socioeconomic indicators and greater deprivation of the health service network in the country. In this cyclical process of multiple influences, it is necessary to consider that the quality of higher education in the health area can interfere with the health system, requiring specific policies in view of the increase in private participation in higher education, the entry of publicly traded institutions, and the presence of large educational groups that work in scale economies^11^. Thus, the low quality of health education is reflected in low quality health services, worsening the situation of small municipalities and areas with deprivation of health services.

In recent years, there has been a significant expansion of higher education in Brazil, which was characterized by low direct public investment and strong stimulus to the performance of the private initiative^9^. This process was induced by favoring the market laws and management of the socioeconomic, health and education policies in force in the country, which promoted the role of the private initiative in the offer of new higher education courses^12^.

The privatization of education was accompanied by public financing policies, with emphasis on the Educational Credit Program, the Higher Education Student Financing Fund (*Fundo de Financiamento ao Estudante do Ensino Superior,* FIES) and ProUni. These policies have been funded with public moneys, but aimed at meeting the interests of some socioeconomic groups, which has generated substantial growth in the offer of courses and private vacancies in health education. Thus, this growth did not benefit the population homogeneously, nor did it meet the needs and vulnerabilities still evident in the country’s population^12^.

The privatization process of higher education in Brazil took place with a questionable and little structured offer of courses or isolated colleges in the regions and cities with the worst social and health indicators of the country. The creation of undergraduate vacancies was not accompanied by investments in the physical infrastructure of the health and education network, nor in the hiring of preceptors with qualified training that allowed for the development of clinical and relational competences for interprofessional care and performance in the students^12^.

The strong stimulus to private higher education was associated with some factors, such as: less competitive student selection processes, with less knowledge potential and accumulation, offering lower quality education, and less qualified faculties and infrastructures^17^ ^,^ ^19^ ^,^ ^26^. These factors can explain part of the differences in the ENADE results between the public and private management undergraduate courses, both among and within the Brazilian regions and states.

In public education, there is an expansion of federal university education, which has been implemented in Brazil since the end of the 1990s: initially, with an increase in vacancies and courses in the existing campuses, and then with the inland-reach and creation of new HEIs. Between 2003 and 2010, the creation of 15 new federal universities in the country stands out, with an 85% increase in the number of campuses. This increase exerted a significant impact on the growth in the number of enrollments, especially in the North (94%) and Northeast (76%) regions^27^. However, such expansion seems to be insufficient to break the exclusionary and unequal historical process in the path of educational access in Brazil. This process is not solved only by entering higher education. The findings show that the regions that historically presented the worst indicators in the educational offer in the country remain with low performance indicators in ENADE.

Despite the expansion of the public sector, growth was still shy when compared to what occurred in private HEIs. From 1995 to 2005, public HEIs had a 10% expansion when compared to 183% of their private counterparts^28^, revealing mismatches in the expansion of the offer and in the organizational and structural characteristics that mark higher education in Brazil.

The commodification of Brazilian higher education with the adoption of market strategies guided by financialization, oligopolization and internationalization^29^ also influenced the expansion of EaD in the country. Therefore, it is necessary to consider the effects of this modality on the quality of the training of health professionals. In this study, greater unsatisfactory performance was verified in EaD than in face-to-face education.

One of the fundamental aspects to define the teaching quality is the faculty; the existence of a qualified faculty (with master’s and PhDs degrees) and the presence of *stricto sensu* graduate programs in the towns where the HEIs are inserted are factors that affected performance in ENADE^30^, especially for the regions that have experienced recent growth in the number of courses. The higher professor/student ratio increases the chances of qualified education^28^. However, what is most observed in the expansion of private education is its disorderly implementation, with a faculty smaller than necessary or with lower qualifications.

The work regime practiced in most of the private HEIs in the country also affects education quality and compromises comprehensive training. Part-time and hourly contracts influence the teaching dynamics and prioritization of the teaching activities to the detriment of research and extension^28^. Thus, the Brazilian higher education counter-reform movement, marked by restrictions in the public budget and stimulus to the expansion of the private sector, led to the strengthening of a marketing logic of the HEIs and to the precariousness of the teaching work.

On the other hand, although ENADE is an important indicator of the higher education courses in Brazil, there are criticisms regarding its exclusive use to measure the quality of training^8^ ^,^ ^26^.

Among its limitations, ENADE is a mandatory test for federal and private HEIs, but it is optional for state and municipal networks. Therefore, the Examination results may not portray the universe of the courses offered in the country. However, the large number of participants and courses evaluated overcomes this limitation, being considered as a national census and representative for the entire country.

Another limitation is that the ENADE evaluation process has focused exclusively on cognitive skills and on the ability to retain contents. ENADE is a complex evaluative activity, is not applied annually for the same undergraduate courses and focuses more on the results than on the process. In addition, in Brazil - a country of continental dimensions - the evaluations have not been considering the cultural differences and socioeconomic and regional inequalities of the country^6^. Both in ENADE and in other exams and tests applied to measure the quality of training, it is observed that some HEIs institute strategies that can camouflage the actual results^31^, such as offering specific preparatory courses and awards for the students to attend and sit for ENADE. In addition, the ENADE results are often used by the media to develop a ranking among the institutions, of a media character of the private institutions^32^.

Despite these reflections, ENADE has been an outstanding standardized method and evaluation indicator of significant validity when presenting the higher education quality level in Brazil over the years. Despite the limitations, the study contributes to a broader analysis of the quality of higher education in health in the country, pointing out regional, structural and organizational differences between the courses.

## Conclusion

The results described the performance level of higher education health students in Brazil as satisfactory, although the proportion of excellence level of this education was still uncommon in the country. Some undergraduate courses presented more unsatisfactory levels than others, especially those of Speech Therapy and Nursing.

Heterogeneity in the quality of this education was deeply observed between the types of public and private institutions, regions of the country, states, population size of the cities and their integration to the MR, indicating that higher education in health in Brazil still concentrates major challenges in order to meet the diversity of the population’s health conditions and needs and of the several organizational characteristics of the health system in the country.

Therefore, these results allow detecting possible deficits related to the HEIs and to the infrastructure of the cities in which they are inserted, enabling greater debate on the training and management of the teaching-learning process in the health area in order to reduce inequalities in the quality of education in different locations of the country. 

The results may support policies and strategies to correct the disparities identified, especially for the worst-performing courses. The study presents diverse evidence that indicates the need to order regulatory measures on the strategies of privatization and financialization of face-to-face education and EaD, which proved to be determinants of the lower quality of higher education in health in Brazil.
